# A fingerprint of 2-[^18^F]FDG radiometabolites – How tissue-specific metabolism beyond 2-[^18^F]FDG-6-P could affect tracer accumulation

**DOI:** 10.1016/j.isci.2023.108137

**Published:** 2023-10-06

**Authors:** Eva-Maria Patronas, Theresa Balber, Anne Miller, Barbara Katharina Geist, Antje Michligk, Chrysoula Vraka, Maximilian Krisch, Nataliya Rohr-Udilova, Arvand Haschemi, Helmut Viernstein, Marcus Hacker, Markus Mitterhauser

**Affiliations:** 1 Department of Biomedical Imaging and Image-guided Therapy, Division of Nuclear Medicine, Medical University of Vienna, Vienna 1090, Austria; 2 Division of Pharmaceutical Technology and Biopharmaceutics, Department of Pharmaceutical Sciences, University of Vienna, Vienna 1090, Austria; 3 Ludwig Boltzmann Institute Applied Diagnostics, Vienna 1090, Austria; 4 Center for Pathobiochemistry and Genetics, Medical University of Vienna, Vienna 1090, Austria; 5 Division of Gastroenterology and Hepatology, Department of Internal Medicine III, Medical University of Vienna, Vienna 1090, Austria; 6 Department of Laboratory Medicine, Medical University of Vienna, Vienna 1090, Austria; 7 University of Vienna, Faculty of Chemistry, Institute of Inorganic Chemistry, Vienna 1090, Austria

**Keywords:** nuclear medicine, radiochemicals, cancer

## Abstract

Studies indicate that the radiotracer 2-[^18^F]fluoro-2-deoxy-D-glucose (2-[^18^F]FDG) can be metabolized beyond 2-[^18^F]FDG-6-phosphate (2-[^18^F]FDG-6-P), but its metabolism is incompletely understood. Most importantly, it remains unclear whether downstream metabolism affects tracer accumulation *in vivo.* Here we present a fingerprint of 2-[^18^F]FDG radiometabolites over time in cancer cells, corresponding tumor xenografts and murine organs. Strikingly, radiometabolites representing glycogen metabolism or the oxPPP correlated inversely with tracer accumulation across all examined tissues. Recent studies suggest that not only hexokinase, but also hexose-6-phosphate dehydrogenase (H6PD), an enzyme of the oxidative pentose phosphate pathway (oxPPP), determines 2-[^18^F]FDG accumulation. However, little is known about the corresponding enzyme glucose-6-phosphate dehydrogenase (G6PD). Our mechanistic *in vitro* experiments on the role of the oxPPP propose that 2-[^18^F]FDG can be metabolized via both G6PD and H6PD, but data from separate enzyme knockdown suggest diverging roles in downstream tracer metabolism. Overall, we propose that tissue-specific metabolism beyond 2-[^18^F]FDG-6-P could matter for imaging.

## Highlights

• Metabolism beyond 2-[^18^F]FDG-6-P is tissue-dependent and increases over time
• Total radiometabolites beyond 2-[^18^F]FDG-6-P in tissues reach up to 39% 120 min p.i
• Specific radiometabolites correlate inversely with total tracer accumulation
• Knockdown of oxPPP enzymes G6PD/H6PD differentially perturbs 2-[^18^F]FDG metabolism

## Graphical abstract

**Figure:**
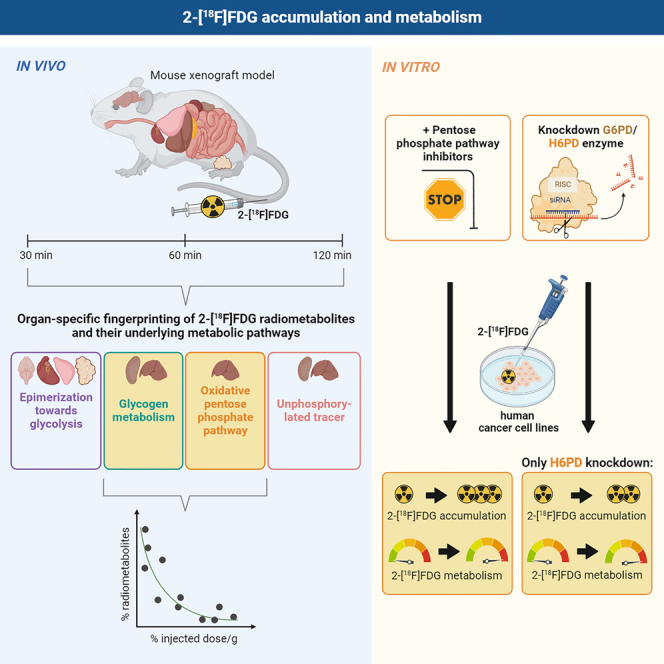

## Introduction

2-[^18^F]FDG can reportedly be metabolized beyond 2-[^18^F]FDG-6-phosphate (2-[^18^F]FDG-6-P), as shown for different cell lines^1^^,^^2^^,^^3^^,^^4^ and individual animal organs or tumor models.^5^^,^^6^^,^^7^^,^^8^ The radiometabolites described include 2-[^18^F]FDG-1-phosphate and 2-[^18^F]fluoro-2-deoxy-6-gluconolactone (2-[^18^F]FD-PGL), indicating that the tracer might be introduced into several pathways such as glycogen metabolism or the pentose phosphate pathway (PPP). Compared to these studies, ^19^F nuclear magnetic resonance imaging (NMR) using the non-radioactive ^19^F-isotopolog of the tracer detected even more metabolites, particularly 2-fluoro-2-deoxy-D-mannose-6-phosphate and further mannose-based metabolites.^9^^,^^10^^,^^11^ This could be due to the use of substantially higher doses, or an analysis at later time-points, as done by Kanazawa et al.^9^

As for 2-[^18^F]FDG accumulation, particular attention has been paid to the pentose phosphate pathway (PPP) during the last years.^12^^,^^13^^,^^14^^,^^15^ The PPP, a carbohydrate metabolic pathway which branches from glucose 6-phosphate (G6P) in parallel to glycolysis, is involved in various cellular processes, as it is a significant source of pentose phosphates and NADPH required for redox balance and the synthesis of biomolecules such as fatty acids. The PPP consists of an oxidative (oxPPP) and a non-oxidative branch, with glucose-6-phosphate dehydrogenase (G6PD) as the first and rate-limiting enzyme of the oxPPP,^16^ converting G6P to 6-phosphogluconolactone. However, there exists a separate PPP in the endoplasmic reticulum (ER),^17^^,^^18^ the compartment where 2-[^18^F]FDG-6-P seems to preferentially accumulate and where its dephosphorylation takes place.^13^^,^^19^^,^^20^ According to literature, the intracellular accumulation of 2-[^18^F]FDG appears to be determined by the oxPPP enzyme hexose-6-phosphate dehydrogenase (H6PD) in the ER.^12^^,^^13^^,^^14^^,^^15^ Unlike G6PD, this enzyme is autosome-linked and performs not only the first step in the oxPPP, as described above, but also the hydrolysis of the gluconolactones thus formed.^17^^,^^18^ H6PD is said to compete with glucose-6-phosphatase (G6Pase) in the ER, thereby preventing the tracer’s dephosphorylation.^13^ In contrast, little is known about the role of the corresponding enzyme G6PD. In literature, G6PD is usually associated with the cytoplasmic oxPPP, while H6PD is often described as ER-exclusive.^15^^,^^18^ However, public protein expression data suggest that neither G6PD nor H6PD are strictly confined to the cytoplasmic or reticular compartment.^21^^,^^22^ Furthermore, it is generally assumed that G6PD cannot process other hexoses than glucose.^23^^,^^24^ However, some studies show the *in vitro* formation of the oxPPP radiometabolite 2-[^18^F]FD-PGL with isolated G6PD enzyme.^2^^,^^25^

While the effect of H6PD expression and activity on 2-[^18^F]FDG accumulation has been thoroughly studied,^12^^,^^13^^,^^14^ the role of G6PD in in this context remains ill-defined. Furthermore, even though metabolism beyond 2-[^18^F]FDG-6-P is undeniable, interorgan or translational differences in 2-[^18^F]FDG metabolism have not been studied in detail, especially over time. Most importantly, it remains unclear if metabolism beyond 2-[^18^F]FDG-6-P is indeed relevant for *in vivo* tracer accumulation and thus routine clinical imaging.

Therefore we aimed to establish a fingerprint of 2-[^18^F]FDG radiometabolites in various murine organs and tumors, as well as to study differences between cultured tumor cells and corresponding tumors *in vivo* to evaluate, in a second step, the impact of downstream metabolism on tracer accumulation. As the oxPPP seems to be highly involved in 2-[^18^F]FDG trapping, we also aimed to further define the role of oxPPP enzymes in regulating tracer accumulation and radiometabolite formation.

## Results

In general, accumulation and metabolism of the radiotracer were evaluated for five different organs and two different types of xenograft tumors of anesthetized and untreated female Fox Chase SCID Beige mice at 30, 60 and 120 min after tracer application. These time-points were based on scan time-points or scan durations commonly used in preclinical static and dynamic 2-[^18^F]FDG μPET studies. While total tracer accumulation was assessed with gamma counter measurements of harvested organs and tumors, metabolism beyond 2-[^18^F]FDG-6-P was evaluated with HPLC measurements of lysed tissues using an anion-exchanger column as shown previously by our group.^2^ Given the recent publications on the importance of the oxPPP for 2-[^18^F]FDG accumulation and our *in vivo* results, the focus *in vitro* was to evaluate the effects of manipulating the PPP. Tracer accumulation and metabolism were investigated upon the addition of oxPPP inhibitors or knockdown of specific oxPPP enzymes in a 2D culture of HT1080 and HT29 cells which were also used to generate xenograft tumors.

### General information about radiometabolites

**Figure 1:**
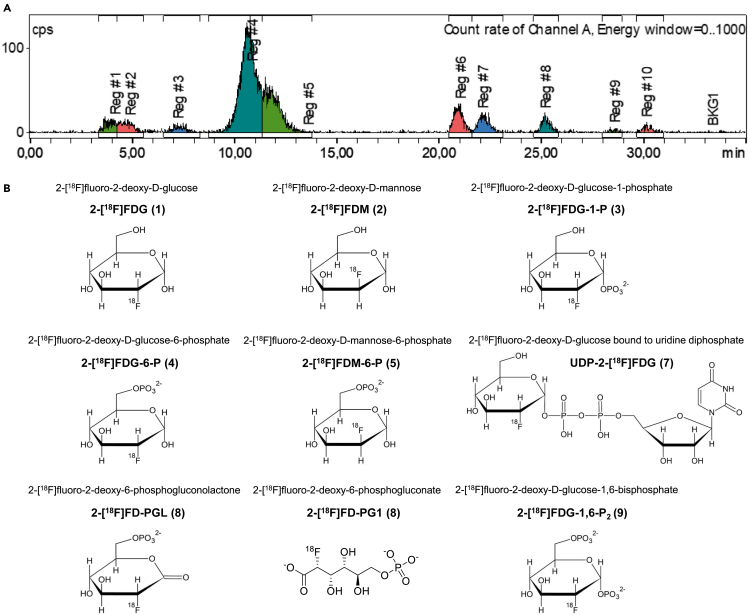
HPLC chromatogram and corresponding chemical structures of the radiotracer and its radiometabolites (A) Representative HPLC chromatogram following organ lysis (mouse lung, 120 min). Regions 6 and 10 depict unidentified radiometabolites. (B) Chemical structures, names, and abbreviations of all discussed radiometabolites.

The following radiometabolites are mentioned in this article: 2-[^18^F]fluoro-2-deoxy-D-mannose (2-[^18^F]FDM), 2-[^18^F]fluoro-2-deoxy-D-glucose-1-phosphate (2-[^18^F]FDG-1-P), 2-[^18^F]fluoro-2-deoxy-D-glucose-6-phosphate (2-[^18^F]FDG-6-P), 2-[^18^F]fluoro-2-deoxy-D-mannose-6-phosphate (2-[^18^F]FDM-6-P), 2-[^18^F]fluoro-2-deoxy-D-glucose bound to uridine diphosphate (UDP-2-[^18^F]FDG), 2-[^18^F]fluoro-2-deoxy-6-phosphogluconolactone (2-[^18^F]FD-PGL), 2-[^18^F]fluoro-2-deoxy-6-phosphogluconate (2-[^18^F]FD-PG1), and 2-[^18^F]fluoro-2-deoxy-D-glucose-1,6-bisphosphate (2-[^18^F]FDG-1,6-P_2_). We previously verified the identity of these radiometabolites,^2^ while we observed one to four additional unidentified radiometabolites (e.g., Regions 6 and 10 in Figure 1A). Importantly, the peak at ∼ 25 min could be 2-[^18^F]FD-PGL, 2-[^18^F]FD-PG1, or a mixture of both, as spontaneous hydrolysis of 2-[^18^F]FD-PGL can occur^6^^,^^26^^,^^27^ and it was not possible to distinguish these two radiometabolites using HPLC and enzymatic *in vitro* synthesis. Therefore, the radiometabolite at ∼ 25 min is specified as 2-[^18^F]FD-PGL/PG1 henceforth in the text.

Figure 1 shows a representative chromatogram, as well as the chemical structures, names and abbreviations of 2-[^18^F]FDG and all radiometabolites discussed in this article.

### Total accumulation of 2-[^18^F]FDG and overall metabolism beyond 2-[^18^F]FDG-6-P are organ-dependent

**Figure 2:**
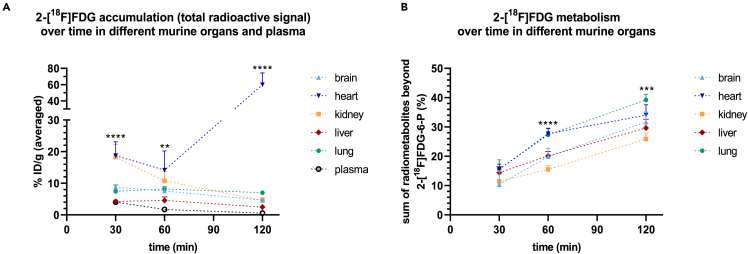
Accumulation of 2-[^18^F]FDG and overall metabolism beyond 2-[^18^F]FDG-6-P are organ-dependent (A) Total radioactivity (percent injected dose per gram, % ID/g) detected in murine organs with *ex vivo* gamma counting 30, 60 and 120 min post injection (n = 3–5) and (B) corresponding overall metabolism beyond 2-[^18^F]FDG-6-P (n = 4–6, n = 3 for lung 120 min). Asterisks highlight significant differences between tissues at each time-point, assessed with one-way ANOVA. Data are represented as mean ± SD (∗∗p ≤ 0.01, ∗∗∗p ≤ 0.001, ∗∗∗∗p ≤ 0.0001).

Total radioactivity (percent injected dose per gram, % ID/g) detected in *ex vivo* measurements was significantly different between murine organs at all tested time-points (n = 3–5). Accumulation was in equilibrium after 30 min for all tissues except for the kidney and plasma, where the activity decreased, and the heart, where the activity increased steadily within the measured time frame (Figure 2A). The latter effect is probably due to isoflurane anesthesia, which is known to enhance 2-[^18^F]FDG accumulation in the heart.^28^^,^^29^ In addition, overall metabolism beyond 2-[^18^F]FDG-6-P at 60 and 120 min, assessed by the sum of derived radiometabolites excluding 2-[^18^F]FDG-6-P, was significantly different between the measured organs (n = 4–6, n = 3 for lung 120 min). At 120 min, lowest radiometabolite levels beyond 2-[^18^F]FDG-6-P were detected in the kidney (26 ± 2.0%), while lung tissue showed the highest levels (39 ± 2.0%) and also the highest slope over 120 min (Figure 2B). A report of the sum of radiometabolites beyond 2-[^18^F]FDG-6-P at all examined time-points is additionally given in Table 1.

**Table 1:** Summary of total radiometabolite levels beyond 2-[^18^F]FDG-6-P for all examined organs and time-points upon tracer injection as assessed with HPLC

	Sum of radiometabolites beyond 2-[^18^F]FDG-6-P (mean % ± SD)					
	30 min		60 min		120 min	
Brain	10 ± 4.8	n = 6	20 ± 2.8	n = 4	32 ± 3.4	n = 6
Heart	16 ± 1.8	n = 4	28 ± 1.8	n = 5	34 ± 3.5	n = 5
Kidney	12 ± 3.3	n = 5	16 ± 1.3	n = 4	26 ± 2.0	n = 4
Liver	14 ± 2.0	n = 4	20 ± 1.5	n = 5	30 ± 3.0	n = 4
Lung	16 ± 3.2	n = 4	27 ± 2.3	n = 4	39 ± 2.0	n = 3

### Analysis of individual radiometabolites reveals distinct tissue-dependent patterns of 2-[^18^F]FDG metabolism

**Figure 3:**
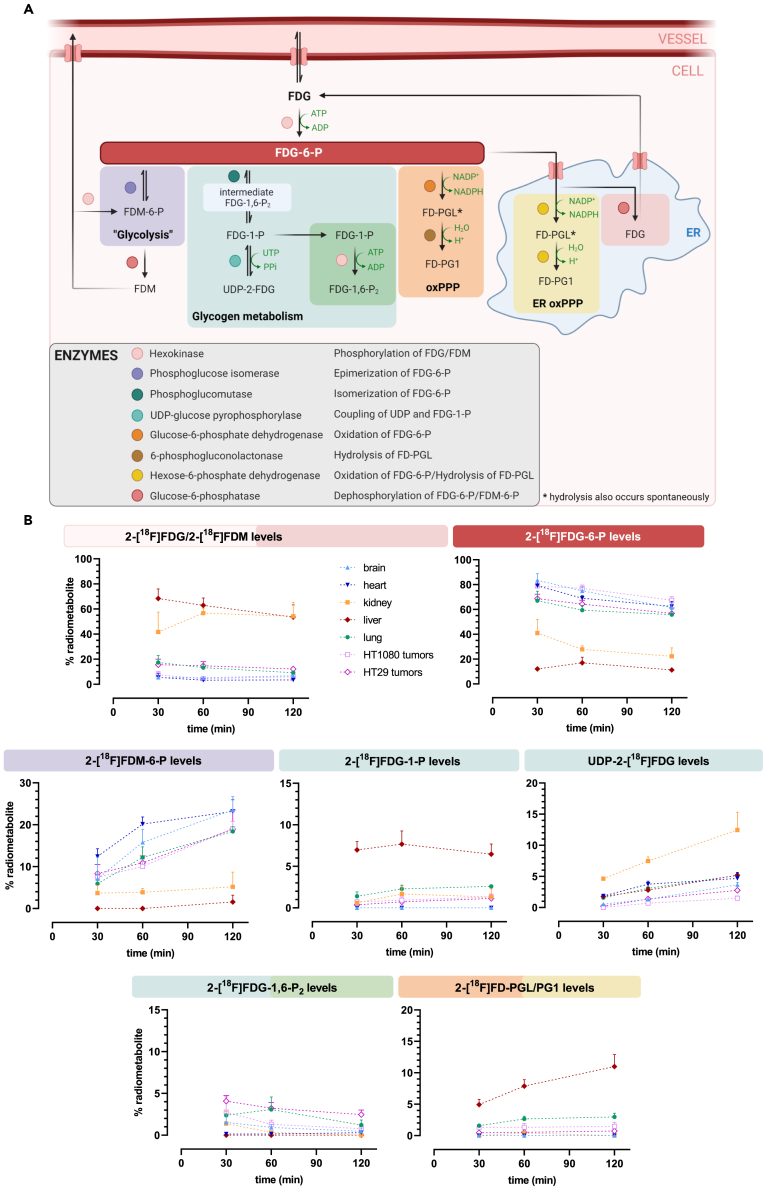
Analysis of individual radiometabolites reveals distinct tissue-dependent patterns of 2-[^18^F]FDG metabolism (A) Proposed pathways of radiometabolite formation in human cells and rodents including the respective enzymes involved, based on our data and literature^2^^,^^4^^,^^5^^,^^8^^,^^10^^,^^12^^,^^13^^,^^25^^,^^30^^,^^31^ (created with BioRender.com, the correct nomenclature has been omitted). ER = endoplasmic reticulum, oxPPP = oxidative pentose phosphate pathway, UTP = uridine triphosphate. Metabolites: FDM = 2-fluoro-2-deoxy-D-mannose, FDM-6-P = 2-fluoro-2-deoxy-D-mannose-6-phosphate, FDG-1-P = 2-fluoro-2-deoxy-D-glucose-1-phosphate, FDG-1,6-P_2_ = 2-fluoro-2-deoxy-D-glucose-1,6-bisphosphate, UDP-2-FDG = 2-FDG bound to uridine diphosphate, FD-PGL = 2-fluoro-2-deoxy-6-phosphogluconolactone, FD-PG1 = 2-[^18^F]fluoro-2-deoxy-6-phosphogluconate. (B) Levels of individual radiometabolites over time in all examined tissues quantified with HPLC (n = 3–6). Data are represented as mean ± SD.

Concerning 2-[^18^F]FDG metabolism in different organs, distinct metabolic patterns were observed (n = 3–6). These patterns as well as proposed pathways of radiometabolite formation are summarized in Figure 3. 2-[^18^F]FDM was sometimes present in *ex vivo* analysis, but without a consistent pattern and 2-[^18^F]FDG and 2-[^18^F]FDM peaks could not be accurately separated for quantitative assessments. Considering that both molecules can exit the cell, we summed the levels of 2-[^18^F]FDG and 2-[^18^F]FDM to form an “unphosphorylated pool.” This pool was highest in the kidney and liver (roughly 60% at both 60 and 120 min), which are known to highly express G6Pase.^32^ 2-[^18^F]FDG-6-P and 2-[^18^F]FDM-6-P levels were lowest in these organs at all investigated time-points. Relevant levels of glycogenic 2-[^18^F]FDG-1-P were only found in the liver, where around 7% were detected at all three time-points. Importantly, this radiometabolite was not observed in the brain and heart. The glycogenic radiometabolite UDP-2-[^18^F]FDG was the main radiometabolite besides 2-[^18^F]FDG-6-P in the kidney, reaching 13 ± 2.9% at 120 min, but remaining below 5% in other tissues. The oxPPP radiometabolite 2-[^18^F]FD-PGL/PG1 remained low in all tissues except for the liver, where it reached 11 ± 1.9% at 120 min. Like 2-[^18^F]FDG-1-P, 2-[^18^F]FD-PGL/PG1 was not detected in the brain and heart. However, as observed for tumors, 2-[^18^F]FDM-6-P was the only relevant radiometabolite besides 2-[^18^F]FDG-6-P in those two organs, reaching 24 ± 3.2% and 23 ± 2.8% at 120 min, respectively. In contrast to cell culture, where it was the main radiometabolite beyond 2-[^18^F]FDG-6-P at 120 min, relatively low levels of 2-[^18^F]FDG-1,6-P_2_ were found *ex vivo*.

In summary, these results indicate tissue-dependent patterns of 2-[^18^F]FDG metabolism. 2-[^18^F]FDM-6-P was the most abundant radiometabolite besides 2-[^18^F]FDG-6-P in the brain, heart, lung and tumors. Glycogenic radiometabolites were prominent in the liver and kidney, while levels of the oxPPP radiometabolite 2-[^18^F]FD-PGL/PG1 were highest in the liver.

### HT1080 and HT29 xenograft tumors differ in 2-[^18^F]FDG accumulation and overall metabolism and show a shifted radiometabolite pattern compared to *in vitro* cell culture

**Figure 4:**
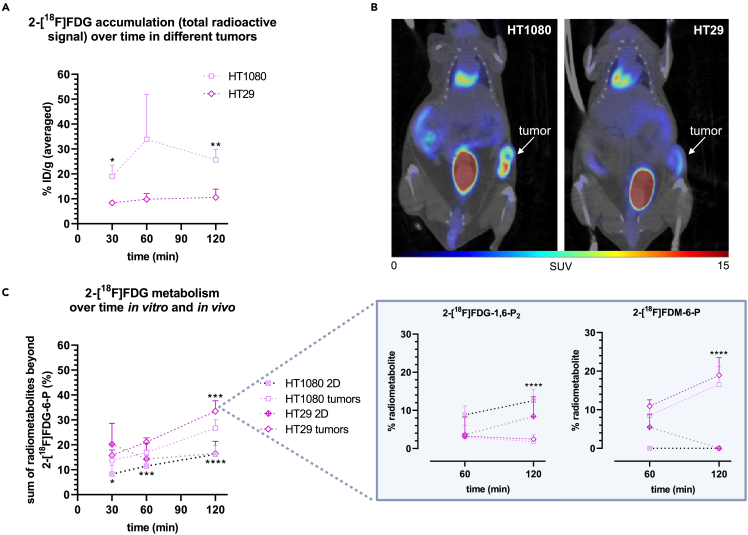
HT1080 and HT29 xenograft tumors differ in 2-[^18^F]FDG accumulation and overall metabolism and show a shifted radiometabolite pattern compared to *in vitro* cell culture (A) Total radioactivity (% ID/g) detected in HT1080 and HT29 tumors over time with *ex vivo* gamma counting (n = 3 for HT1080, n = 4 for HT29). Asterisks report significant differences between the two tumor types at the respective time-point (unpaired t-test). (B) Corresponding representative μPET/CT images (frame 55 and 65 min averaged). (C) Comparison between 2-[^18^F]FDG metabolism *in vitro* and *in vivo* (n = 4–12). Asterisks show significant differences between 2D culture and tumors for each cell line at each time-point (unpaired t-test). The box shows most relevant radiometabolites besides 2-[^18^F]FDG-6-P (time-point 120 min assessed with one-way ANOVA). Data are represented as mean ± SD (∗p ≤ 0.05, ∗∗p ≤ 0.01, ∗∗∗p ≤ 0.001, ∗∗∗∗p ≤ 0.0001).

Higher total radioactivity was detected in the more aggressive HT1080 tumors compared to HT29 tumors as determined by *ex vivo* gamma counting (n = 3–4) (Figure 4A), which is in line with the representative μPET/CT images (Figure 4B). Unexpectedly, a comparison of the *in vitro* and *in vivo* fingerprint of 2-[^18^F]FDG metabolism indicated that 2-[^18^F]FDG metabolism beyond 2-[^18^F]FDG-6-P was around 1.5-fold (HT1080) and 2-fold (HT29) higher at 120 min *in vivo* (n = 4–9) (Figure 4C). Higher levels of unphosphorylated tracer, that can potentially exit the cell, were found in HT29 tumors (data not shown) at all studied time-points, which agreed with accumulation data. In contrast to HT1080 and *in vivo* data, overall metabolism in HT29 did not increase over time *in vitro* (Figure 4C). In general, the radiometabolite profile was inherently different between *in vitro* cell culture and corresponding tumors (n = 4–12): *in vitro,* the most abundant radiometabolite besides 2-[^18^F]FDG-6-P at 120 min was 2-[^18^F]FDG-1,6-P_2_, reaching a maximum of 13 ± 3.0% of total radiometabolites beyond 2-[^18^F]FDG-6-P in HT1080 and 8.4 ± 5.1% in HT29 cells. However, *in vivo* it was 2-[^18^F]FDM-6-P with levels up to 17 ± 4.6% and 19 ± 4.5%, respectively (Figure 4C, box).

### Radiometabolites of glycogen metabolism and the oxidative pentose phosphate pathway correlate with total radioactivity in tissues and with each other

**Figure 5:**
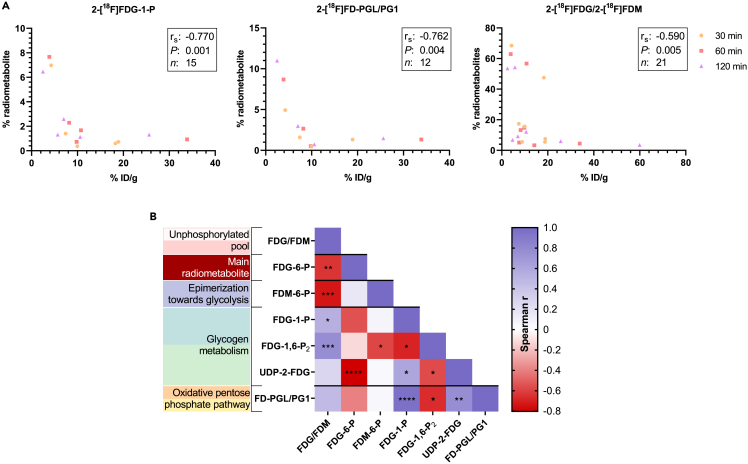
Radiometabolites of glycogen metabolism and the oxPPP correlate with total radioactivity in tissues and with each other (A) Spearman correlation between the levels of 2-[^18^F]FDG-1-P, 2-[^18^F]FD-PGL/PG1, 2-[^18^F]FDG/2-[^18^F]FDM (unphosphorylated pool) and percent injected dose per gram (% ID/g) tissue. (B) Heatmap of correlations between radiometabolites (n = 9–21). Asterisks report statistical significance: ∗p ≤ 0.05, ∗∗p ≤ 0.01, ∗∗∗p ≤ 0.001, ∗∗∗∗p ≤ 0.0001; the correct nomenclature has been omitted in (B).

The radiometabolite levels were correlated with total tissue radioactivity measured via gamma counter (% ID/g) at the time-points 30, 60 and 120 min. Comparing all measured tissues and time-points, the radiometabolites 2-[^18^F]FD-PGL/PG1, 2-[^18^F]FDG-1-P, and the unphosphorylated pool correlated inversely with the measured % ID/g (n = 12, r_s_ = −0.762, p = 0.004; n = 15, r_s_ = −0.770, p = 0.001; n = 21, r_s_ = −0.590, p = 0.005, respectively) (Figure 5A). Considering that unphosphorylated tracer can leave the cell, the observed inverse correlation between the unphosphorylated pool and total 2-[^18^F]FDG accumulation can be considered as proof of principle. In contrast, the sum of all radiometabolites beyond 2-[^18^F]FDG-6-P did not correlate with tracer accumulation, indicating their selective function in regulating 2-[^18^F]FDG metabolism. As a cross-check, radiometabolite levels were further correlated with corresponding % ID/cc values of the five dynamic scans by defining volumes of interest in the organs (the approach is specified in the STAR methods section). This cross-check revealed almost identical correlations (data not shown).

To reveal potential relationships between radiometabolites, their levels were correlated with each other, and the Spearman r was plotted in a heatmap (Figure 5B). A strong correlation was identified between the oxPPP radiometabolite 2-[^18^F]FD-PGL/PG1 and the glycogenic intermediates 2-[^18^F]FDG-1-P and UDP-2-[^18^F]FDG (r_s_ = 0.953, p ≤ 0.0001 and r_s_ = 0.769, p = 0.003, respectively). As expected, this analysis also revealed a correlation between 2-[^18^F]FDG-1-P and UDP-2-[^18^F]FDG (r_s_ = 0.599, p = 0.018). UDP-2-[^18^F]FDG was the only radiometabolite that strongly correlated inversely with 2-[^18^F]FDG-6-P (r_s_ = −0.804, p ≤ 0.0001), while a trend was also observed with 2-[^18^F]FDG-1-P (r_s_ = −0.550, p = 0.064). The unphosphorylated pool correlated inversely with 2-[^18^F]FDG-6-P and 2-[^18^F]FDM-6-P (r_s_ = −0.643, p = 0.004 and r_s_ = −0.736, p = 0.001), while no or a positive correlation was observed with other radiometabolites.

### The oxidative pentose phosphate pathway inhibitors dehydroepiandrosterone and G6PDi-1, but not carbenoxolone enhance 2-[^18^F]FDG accumulation and metabolism

**Figure 6:**
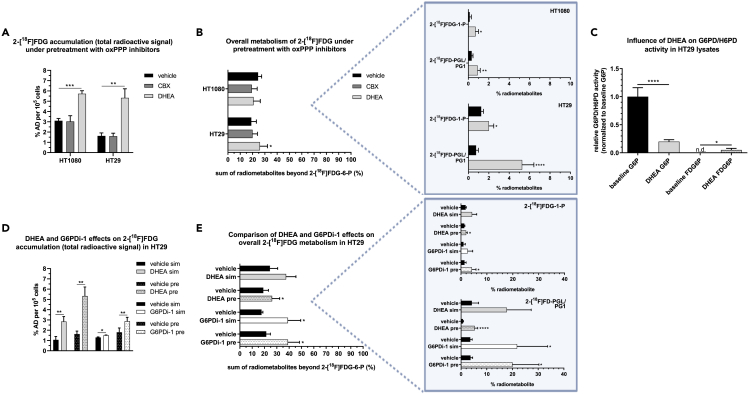
The oxPPP inhibitors DHEA and G6PDi-1, but not CBX enhance 2-[^18^F]FDG accumulation and metabolism (A and B) Effects of the inhibitors CBX or DHEA on total tracer accumulation (n = 3) and metabolism (n = 4–7) 1 h post 2-[^18^F]FDG addition (% AD = percent applied dose). (C) Relative G6PD/H6PD activity in HT29 lysates with or without DHEA using G6P or FDG6P as substrate (lysates from two different days). (D and E) Comparison of DHEA and G6PDi-1 effects on tracer accumulation (n = 3) and metabolism (n = 3–7), applying the inhibitors 1 h before (pre), or simultaneous with 2-[^18^F]FDG (sim). Boxes show relevant radiometabolite changes. In all parts of the graph, asterisks highlight significant differences between treatment and vehicle groups (unpaired t-test, ∗p ≤ 0.05, ∗∗p ≤ 0.01, ∗∗∗p ≤ 0.001, ∗∗∗∗p ≤ 0.0001). Data are represented as mean ± SD.

To test whether the first step of the oxPPP - catalyzed by G6PD/H6PD - can influence 2-[^18^F]FDG accumulation and metabolism, we chose a pharmacological approach and pretreated cells with G6PD/H6PD inhibitors. Unexpectedly, only dehydroepiandrosterone (DHEA) increased 2-[^18^F]FDG accumulation in HT1080 and HT29 cells by approximately two- and 3-fold 1 h after tracer application, respectively (n = 3), and also downstream metabolism from 19 ± 4.2% to 26 ± 6.1% in HT29 cells, while carbenoxolone (CBX) showed no effect (n = 4–7) (Figures 6A and 6B). As for specific radiometabolites, DHEA increased 2-[^18^F]FDG-1-P and 2-[^18^F]FD-PGL/PG1 levels in both cell lines, but the levels were generally low in HT1080 (n = 4–6, Figure 6B, box).

To ensure the inhibitory function of DHEA, an *ex vivo* G6PD/H6PD activity assay with HT29 lysates was performed. As expected, DHEA decreased the specific activity by ∼ 80% using G6P as a substrate. No baseline activity was observed with FDG6P as substrate, but low specific activity was observed in the presence of DHEA (Figure 6C).

To validate this observation, DHEA effects were compared to an alternative inhibitor, G6PDi-1, using HT29 cells. Both inhibitors increased 2-[^18^F]FDG accumulation, when they were added 1 h before or simultaneous with 2-[^18^F]FDG (n = 3), however, DHEA effects were more pronounced at tested concentrations (Figure 6D). As for 2-[^18^F]FDG downstream metabolism, G6PDi-1 reproduced the enhancing effects of DHEA on overall metabolism and specifically on 2-[^18^F]FDG-1-P and 2-[^18^F]FD-PGL/PG1 (n = 3–7) (Figure 6E). Thereby the data suggested an inverse correlation of G6PD/H6PD activity with 2-[^18^F]FDG accumulation and showed an unexpected increase of the oxPPP radiometabolite in the presence of these inhibitors.

### Separate knockdown of H6PD and G6PD has differential effects on 2-[^18^F]FDG accumulation and metabolism

**Figure 7:**
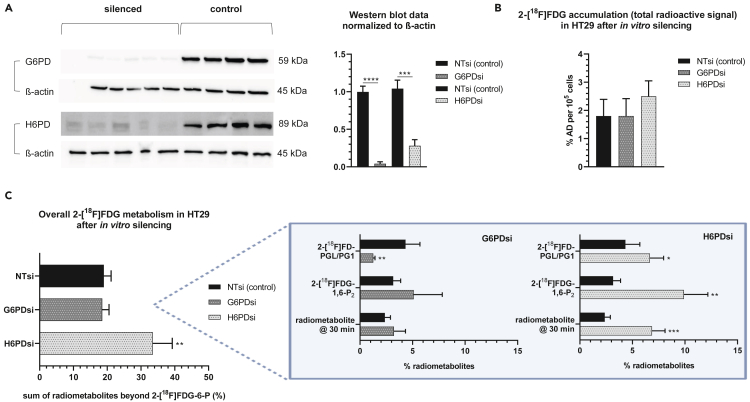
Separate knockdown of H6PD and G6PD has differential effects on 2-[^18^F]FDG accumulation and metabolism (A) WB of HT29 cells treated with siRNA for the respective enzyme or non-targeting siRNA (NTsi) (samples from different experiments). (B and C) Effects of G6PD/H6PD knockdown on 2-[^18^F]FDG accumulation (B) and metabolism (n = 4 each) (C), the box showing relevant radiometabolite changes. % AD = % applied dose. Asterisks mark significant differences between knockdown groups (H6PDsi or G6PDsi) and NTsi (control) (unpaired t-test, ∗p ≤ 0.05, ∗∗p ≤ 0.01, ∗∗∗p ≤ 0.001, ∗∗∗∗p ≤ 0.0001). Data are represented as mean ± SD.

In order to analyze the individual contributions of G6PD and H6PD to observed effects, we selectively silenced these enzymes by siRNA, which was confirmed via Western blot (WB) (Figure 7A). A selective knockdown of H6PD in HT29 cells resulted in a non-significant increase of total 2-[^18^F]FDG accumulation from 1.8 ± 0.59 to 2.5 ± 0.55% applied dose (% AD) per 10^5^ cells (n = 4) compared to control, similar to the effects of DHEA or G6PDi-1. However, the accumulation in G6PD knockdown cells was comparable to control (1.8 ± 0.62% AD, Figure 7B). Interestingly, following G6PD knockdown, the oxPPP radiometabolite 2-[^18^F]FD-PGL/PG1 was considerably reduced from 4.3 ± 1.4% to 1.3 ± 0.17% (Figure 7C, box). While it had only a small influence on the oxPPP radiometabolite, knockdown of H6PD significantly increased overall metabolism beyond 2-[^18^F]FDG-6-P, particularly levels of 2-[^18^F]FDG-1,6-P_2_ and the subsequent, unidentified radiometabolite at minute 30 of the HPLC run (n = 4, Figure 7C). Based on Fedders et al.,^4^ this peak could be 2-[^18^F]fluoro-2-deoxy-D-glucuronic acid. These results indicate a divergent role of the oxPPP enzymes G6PD and H6PD in 2-[^18^F]FDG accumulation and metabolism.

## Discussion

In general, *in vivo* metabolism beyond 2-[^18^F]FDG-6-P reached up to 28% at 60 min and 39% at 120 min, reinforcing the opinion that tracer metabolism, at least in prolonged studies, should not be dismissed and might be relevant for accurate signal quantification in imaging^6^^,^^33^ (Figure 2). Concerning tissue-specific patterns, UDP-2-[^18^F]FDG levels were high in the kidney, an organ with usually low glycogen levels building glycogen under hyperglycemic conditions.^34^ We hypothesize that this is due to hyperglycemia, which is reportedly induced by isoflurane anesthesia.^35^ Radiometabolite data of the liver solidifies the assumption, although the other glycogenic radiometabolite 2-[^18^F]FDG-1-P was more prominent here. Given the proximity of the two peaks in HPLC, we acknowledge that what we described as UDP-2-[^18^F]FDG might also be or include the epimerized form UDP-2-[^18^F]fluoro-2-deoxy-D-galactose, as postulated by Fedders et al.^4^ The most abundant radiometabolite in the liver was 2-[^18^F]FD-PGL/PG1, which is consistent with literature describing highest PPP activity in organs performing lipid and steroid synthesis, such as the liver,^36^ and also reflects previously reported ex *vivo* data^5^^,^^6^ (Figure 3B). As for xenograft tumors, the observed radiometabolites were different from those reported by Suolinna et al., Haaparanta et al. Kaarstad et al., who described nucleotide-bound 2-[^18^F]FDG/UDP-2-[^18^F]FDG^8^^,^^25^, or 2-[^18^F]FD-PGL/PG1 and 2-[^18^F]FDG-1,6-P_2_^7^ as only radiometabolites, respectively. However, different rodents, different types of (non-continuous) anesthesia, other tumor models, as well as other tissue processing and HPLC methods were used.

2-[^18^F]FDM-6-P was the only other quantitatively relevant radiometabolite besides 2-[^18^F]FDG-6-P in all tissues except for the liver and kidney, which have a different physiological function as excretory organs (Figure 3B). Although downstream metabolism was much higher than in our study, Southworth et al., using 150 mg/kg FDG and ^19^F NMR, also demonstrated this large difference between 2-[^18^F]FDM-6-P levels in the rat brain and heart compared to the liver and kidney.^10^ It was suggested in several publications^37^^,^^38^^,^^39^ and later verified by O’Connell et al.^40^ that phosphoglucose isomerase (PGI), the glycolytic enzyme forming fructose-6-phosphate, can reversibly produce 2-[^18^F]FDM-6-P from 2-[^18^F]FDG-6-P via an enol intermediate. This reaction, followed by dephosphorylation as for 2-[^18^F]FDG-6-P appears to be the source of 2-[^18^F]FDM.^40^ Interestingly, except for our preceding study, were we successfully produced 2-[^18^F]FDM-6-P through PGI addition,^2^ 2-[^18^F]FDM-6-P was not described in other studies specifically using the radioactive tracer.^1^^,^^3^^,^^4^^,^^5^^,^^6^^,^^7^^,^^8^^,^^25^ Given the function of PGI, it has been discussed if 2-[^18^F]FDG-6-P epimerization to 2-[^18^F]FDM-6-P could be used as readout for glycolytic activity.^37^^,^^41^ Comparing 2-[^18^F]FDM-6-P levels in 2D cell culture and corresponding tumors, 2-[^18^F]FDG-6-P epimerization is much more pronounced in the latter (Figure 4C). This is in line with Murakami et al., showing that fructose-6-phosphate formation by PGI, the first step toward glycolysis, is much higher in corresponding tumors.^42^ We believe that considering 2-[^18^F]FDM-6-P formation as a general indicator of glycolysis might be an oversimplification. However, it might give an indication of how much the metabolic flux is shifted toward glycolysis in a first step, since 2-[^18^F]FDG-6-P can be either epimerized to 2-[^18^F]FDM-6-P, isomerized to 2-[^18^F]FDG-1-P (→ glycogen metabolism), or oxidized to 2-[^18^F]FD-PGL (→ PPP). Interestingly, while there was no connection between 2-[^18^F]FDM-6-P levels and total tracer accumulation, we found an inverse correlation between the unphosphorylated pool and the radiometabolites 2-[^18^F]FDG-6-P and 2-[^18^F]FDM-6-P. This hints at a link between 2-[^18^F]FDG “trapping” and the tracer being directed toward glycolysis, indicated by 2-[^18^F]FDM-6-P formation.

The inverse correlation between 2-[^18^F]FDG-1-P and 2-[^18^F]FD-PGL/PG1 levels and total 2-[^18^F]FDG accumulation suggests that a) 2-[^18^F]FDG metabolism via glycogen metabolism and the oxPPP could influence tracer accumulation *in vivo*, or b) that an enhanced activity of glycogen metabolism or the oxPPP rewires the cells’ metabolism, causing altered 2-[^18^F]FDG accumulation through changes in the activity of other enzymes or transporters (Figure 5A). Interestingly, we also observed a correlation between the oxPPP radiometabolite 2-[^18^F]FD-PGL/PG1 and the glycogenic radiometabolites 2-[^18^F]FDG-1-P and UDP-2-[^18^F]FDG in *ex vivo* analysis (Figure 5B). This link was consistent *in vitro*, where increased 2-[^18^F]FD-PGL/PG1 levels following inhibitor treatment were accompanied by increased 2-[^18^F]FDG-1-P levels (Figure 6). Taken together, these data underscore the close link between glycogen metabolism and the PPP previously described for macrophages and cyanobacteria.^43^^,^^44^

In our *in vitro* experiments to assess the impact of oxPPP activity on 2-[^18^F]FDG accumulation and metabolism, CBX pretreatment did not alter 2-[^18^F]FDG accumulation, which is in contrast to previously published *in vitro* experiments showing decreased accumulation using this indirect H6PD inhibitor^12^ (Figure 6A). Instead, DHEA and G6PDi-1 enhanced both tracer accumulation and metabolism beyond 2-[^18^F]FDG-6-P in a similar way (Figures 6A, 6B, 6D, 6E). The stronger effect of DHEA on tracer accumulation could be due to the reported stimulation of glucose transporter translocation to the cell membrane.^45^ As the non-steroidal G6PDi-1 has been shown to be a more potent and specific G6PD inhibitor compared to the nonspecific hormone DHEA,^46^ the observed effects could indeed be partly attributable to a specific interaction with the oxPPP. Interestingly, the data of the *ex vivo* enzyme activity assay using saturating concentrations of either G6P or FDG6P clearly demonstrated a different regulatory effect of DHEA on substrate usage of G6PD/H6PD (Figure 6C). The observed higher specific activity of G6PD/H6PD for FDG6P upon DHEA treatment could serve as the potential explanation of the unexpected increase in oxPPP radiometabolite levels although oxPPP activity is repressed. Together these data indicate that the activity of G6PD/H6PD might have effects on both 2-[^18^F]FDG accumulation and metabolism, however, their selective contribution remained to be resolved. For all used inhibitors it is unclear whether only G6PD, H6PD, or both enzymes are targeted. At least the most popular G6PD inhibitor DHEA seems to also inhibit 11β-hydroxysteroid dehydrogenase,^47^ the same mechanism by which CBX indirectly blocks H6PD activity.^48^ To further understand these effects, we separately knocked down both of the first oxPPP enzymes, G6PD and H6PD (Figure 7). Interestingly, only H6PD knockdown enhanced downstream metabolism and also slightly increased 2-[^18^F]FDG accumulation by trend. Therefore, we hypothesized that the enhanced metabolism and accumulation of the tracer observed with DHEA/G6PDi-1 might be mainly due to an interaction with H6PD. Unfortunately, *in vivo* knockdown experiments to further analyze G6PD and H6PD effects were unsuccessful (Figure S3), which might have been due to the density of HT29 tumors or the unmet need for the prolonged repetitive injection of siRNA as reported in other studies.^49^^,^^50^ While our data suggest that H6PD plays a role in 2-[^18^F]FDG accumulation and metabolism as reported in previous studies,^12^^,^^13^^,^^14^^,^^15^ the effect on accumulation upon enzyme knockdown was small and the opposite to what was shown by Marini et al.^12^ The cell type and cell culture methods, including nutrient availability, can have a profound impact on the intracellular metabolic state,^51^^,^^52^ influencing the uptake and downstream pathways of 2-[^18^F]FDG utilization. Higher glucose concentrations are typically associated with PPP stimulation, but this regulation is complex and can vary significantly based on factors such as the expression of glucose transporters, metabolic enzyme expression, and the cell’s signaling state. Instead of glucose-free cell culture medium, which could *per se* induce metabolic adaptation, we used medium with physiological glucose concentration. This might be one of the reasons for the divergent results regarding CBX effects, as mentioned above, and H6PD knockdown. Furthermore, although it is believed that 2-[^18^F]FDG metabolism via the oxPPP is confined to H6PD, we observed significantly reduced levels of 2-[^18^F]FD-PGL/PG1 upon specific G6PD knockdown (Figure 7C). This agrees with previous *in vitro* studies where 2-[^18^F]FD-PGL was successfully synthesized using isolated G6PD.^2^^,^^25^ While we observed a clear inhibition of G6PD/H6PD activity in response to DHEA addition *in vitro*, the increase in 2-[^18^F]FD-PGL/PG1 levels in cells suggests a more intricate regulatory network at play. This may involve feedback inhibition on downstream reactions or secondary regulatory control on upstream enzymes, potentially leading to an increase in substrate availability and, consequently, increased downstream product formation. Another, less likely possibility is the existence of an unknown secondary pathway for 2-[^18^F]FDG entry at this step, such as oxidation to form fluorinated gluconate, which could be phosphorylated to form 2-[^18^F]FD-PG1 independently of G6PD/H6PD.^53^^,^^54^

To put our results in a clinical context, we used the Stanford 2021 PRECOG database to examine G6PD/H6PD gene expression in tumors in relation to patient survival.^55^ We observed that overall, higher H6PD expression is associated with longer survival, while higher G6PD expression is linked to shorter survival across different types of cancer (Table S2). Considering that high 2-[^18^F]FDG accumulation is generally associated with poor prognosis,^56^^,^^57^^,^^58^^,^^59^ this provides an interesting bridge to our finding that higher oxPPP activity inversely correlates with tracer accumulation *in vivo*, likely influenced by H6PD.

In summary, we present the extensive evaluation of 2-[^18^F]FDG metabolism over time in different cells and tissues, revealing a distinct, tissue-dependent metabolic pattern. Regarding 2-[^18^F]FDG metabolism via the oxPPP, our data propose that both G6PD and H6PD affect downstream tracer metabolism in different ways. Most importantly, our data suggest that the metabolic flux of 2-[^18^F]FDG into the oxPPP or glycogen metabolism or an altered activity of these specific pathways could impact tracer accumulation and hence imaging. These data challenge our current simplistic view on the mechanistics and meaning of 2-[^18^F]FDG accumulation and might provide a basis for a better understanding of less 2-[^18^F]FDG avid tissues beyond glucose transporter or hexokinase expression. Furthermore, as already suggested by Kaarstad et al. 20 years ago, the existence of radiometabolites different from 2-[^18^F]FDG-6-P “*may have implications for the interpretation of estimated kinetic rate constants in terms of the enzymatic processes.*”^7^ There is no doubt that a simplified quantification of 2-[^18^F]FDG images using standard uptake values is sufficient in many cases. However, given the extensive metabolism beyond 2-[^18^F]FDG-6-P already at 60 min post injection in some organs, we suggest that extended kinetic models may be needed in future dynamic preclinical studies for correct absolute quantification and representation of underlying (patho)physiological processes. Finally, the potential quantification of radiometabolites derived from specific glucose metabolic pathways opens the possibility to better characterize basic tissue metabolism, tumor heterogeneity, or treatment effects in a preclinical setting, complementing other approaches such as metabolomics or genomics.

### Limitations of the study

We used immunodeficient mice to evaluate tracer metabolism of HT1080 and HT29 tumors. However, the mouse model used has intact dendritic cells and macrophages and we did not investigate their contribution to tracer accumulation and metabolism in the tumor. We also cannot exclude that the subcutaneous tumors affected the metabolism of healthy mouse organs. In another study, tumor growth induced a metabolic shift in non-involved organs on day 6 and day 39 post inoculation in athymic nude mice.^60^ Although the effects were much more pronounced at the later time-point and a different mouse strain and application site was used, the tumor size in our study was closer to that on day 39 in the other study. In addition to that, we continuously anesthetized mice until sacrificing to normalize conditions and make scan data comparable to other *ex vivo* data. As mentioned above, isoflurane anesthesia causes hyperglycemia in mice especially at levels higher than 1.5% v/v,^35^ potentially altering the metabolic pattern. In that context, a shift in the FDG/FDM ratio due to phenobarbital anesthesia was previously reported in a ^19^F NMR study.^37^ Therefore, comparability with other experimental approaches using conscious animals may be limited. Lastly, we acknowledge that the peaks of the radiometabolites 2-[^18^F]FDG-6-P and 2-[^18^F]FDM-6-P were not baseline separated in HPLC analysis, which causes a quantification bias. However, the peaks were always integrated in the same manner by the same person using a perpendicular drop.

## STAR★Methods

### Key resources table

**Table:** 

REAGENT or RESOURCE	SOURCE	IDENTIFIER
**Antibodies**		

anti-G6PD	Abcam	Cat# ab210702; RRID:AB_2923527
anti-H6PD	Abcam	Cat# ab170895; RRID:AB_2864314
anti-H6PD	Proteintech	Cat# 15255-1-AP; RRID:AB_10642699
anti-β-actin	Abcam	Cat# ab8227; RRID:AB_2305186
Goat anti-Rabbit IgG	ThermoFisher	Cat# A16104; RRID:AB_2534776

**Chemicals, peptides, and recombinant proteins**		

2-[^18^F]FDG	in-house production, formulated for patient use	N/A
Matrigel	Sigma-Aldrich	Cat# E6909
Modified Eagle Medium	Gibco^TM^	Cat# 10370-047
Roswell Park Memorial Medium	Gibco^TM^	Cat# 21875-034
L-glutamine	Gibco^TM^	Cat# 25030-024
Fetal bovine serum	Gibco^TM^	Cat# 10270-106
Opti-MEM	Gibco^TM^	Cat# 31985070
DharmaFECT transfection reagent 1	Horizon	Cat# T-2001-03
In vivo-jetPEI®	Polyplus	Cat# 101000040
Dehydroepiandrosterone	Sigma-Aldrich	Cat# D4000
Carbenoxolone	Sigma-Aldrich	Cat# C4790
G6PDi-1	Kindly provided by the Rabinowitz-Lab	Cat# SML2980
Glucose-6-phosphate	Sigma-Aldrich	Cat# G7879
2-fluoro-2-deoxy-D-glucose-6-phosphate	Biosynth® Carbosynth	Cat# MD94505

**Critical commercial assays**		

Pierce™ BCA Protein Assay Kit	ThermoFisher	Cat# 23225

**Experimental models: Cell lines**		

HT1080 cell line	ATCC	CCL-121™; RRID:CVCL_0317
HT29 cell line	ATCC	HTB-38™; RRID:CVCL_0320

**Experimental models: Organisms/strains**		

Fox Chase SCID Beige mice (female)	Charles River	CB17.Cg-Prkdc^scid^Lyst^bg-J^/Crl; RRID:IMSR_CRL:250

**Oligonucleotides**		

ON-TARGETplus siRNA pool G6PD ACAGAUACAAGAACGUGAA, CCGUGUA CACCAAGAUGAU, CAGAUAGGCUGGAA CCGCA, AUUCACGAGUCCUGCAUGA	Horizon	Cat# L-008181-02-0005
ON-TARGETplus siRNA pool H6PD CGUCUGUUAUAAAGCGUUA, CGUGGUGG GCUGAGGUUAA, GCGGGUUGUCCUUGA GAAA, UGGACGAGAGAGUGGGCUA	Horizon	Cat# L-004692-01-0005
ON-TARGETplus siRNA pool non-targeting CCGCAGGGCUCAUGAGUAU, GGACAAACA CCCAUGAACA, AGGAACAAACGUUGACUUA, CCAAAUCUCGUGAUGAAUC	Horizon	Cat# D-001810-10-20
G6PD siRNA “No. 19” (*in vivo* grade) CCGUGUACACCAAGAUGAU	Horizon	Cat# CTM-733527
G6PD siRNA “No. 20” (*in vivo* grade) CAGAUAGGCUGGAACCGCA	Horizon	Cat# CTM-733528
H6PD siRNA “No. 10” (*in vivo* grade) CGUGGUGGGCUGAGGUUAA	Horizon	Cat# CTM-733521
H6PD siRNA “No. 11” (*in vivo* grade) GCGGGUUGUCCUUGAGAAA	Horizon	Cat# CTM-733525
Non-targeting siRNA (*in vivo* grade) GGACAAAC ACCCAUGAACA	Horizon	*in vivo* grade Cat# J-016083-06
Cy5.5 labeled non-targeting siRNA (*in vivo* grade) GGACAAACACCCAUGAACA	Horizon	Cy5.5 labeled *in vivo* grade Cat# J-016083-06

**Software and algorithms**		

PMOD	PMOD Technologies LLC	Version 3.8; RRID:SCR_016547
Fiji	N/A	RRID:SCR_002285
GraphPad Prism	GraphPad	Version 7.03, RRID:SCR_002798
SPSS statistics	IBM	Version 27, RRID:SCR_019096
Gina X HPLC software	Elysia Raytest	Version 10.4

**Other**		

Partisil™ 10 SAX column (250 mm × 4.6 mm)	Supelco Analytical	Cat# 50193-U
Wizard^2^3 2480 automatic gamma counter	PerkinElmer	N/A
Inveon® μPET/SPECT/CT	Siemens	N/A
HPLC system	Shimadzu	N/A
Ramona∗ radioactivity-HPLC flow detector	Elysia Raytest	N/A

### Resource availability

#### Lead contact

Further information and requests for resources and reagents should be directed to the lead contact, Markus Mitterhauser (markus.mitterhauser@univie.ac.at).

#### Materials availability

This study did not generate new unique reagents.

### Experimental model and study participant details

#### Mice

For *in vivo* experiments with the radiotracer 9- to 14-week-old (time of sacrificing) female Fox Chase SCID Beige mice (Charles River, RRID:IMSR_CRL:250) were used. This mouse strain has a severe immunodeficiency, characterized by absent B- and T-lymphocytes, and defective natural killer cells. However, dendritic cells and macrophages are present. The animal experiments were approved by the Federal Ministry of Education, Science and Research and conducted in accordance with the Austrian laws for animal protection.

Until the day of the experiment, the animals were fed *ad libitum* with LASQCdiet Rod16-A (LASvendi). They were housed in Tecniplast Green Line cages with Smart Flow (up to 8 animals per cage), using LASbedding PG2 (LASvendi) and Sizzle-Pet, as well as egg boxes, bio-huts, nesting sheets, and wooden chew as enrichment (all autoclaved). The night-day cycle was 12/12 h. On the day of the experiment, mice weighed between 16.5 and 23.3 g.

#### Cell culture

The human cancer cell lines HT29 (colorectal adenocarcinoma) and HT1080 (fibrosarcoma) were cultured in Roswell Park Memorial Institute medium and Modified Eagle Medium (MEM), respectively, substituted with 2 mM glutamine and 10% fetal bovine serum (all Gibco, ThermoFisher) under standardized conditions (humidified atmosphere, 37°C, 5% CO_2_). The cells were generous gifts of other departments as stated elsewhere,^2^ but were originally obtained from ATCC (RRID:CVCL_0320 and RRID:CVCL_0317). HT1080 cells have been authenticated by the multiplex human cell line authentication test based on single nucleotide polymorphism typing.

### Method details

#### Radiotracer

2-[^18^F]FDG was synthesized in-house at the Department of Biomedical Imaging and Image-guided Therapy, University Hospital Vienna, and formulated for patient use.

#### Animal experiments

Approximately two weeks after arrival, the animals were subcutaneously injected with 2 × 10^6^ tumor cells in phosphate buffered saline (PBS) and 20% matrigel (Sigma-Aldrich) above the right flank. The desired tumor size of 50–150 mm^3^ (length × (width)^2^ × 0.5) was reached after around 10 days for HT29 and after around 7 days for HT1080. On the day of the experiment, animals were fasted for 4 h. Then, the mice were injected with 2-[^18^F]FDG via the tail-vein, warmed and kept under 1–2% isoflurane anesthesia until sacrificing. In general, animals received 14–22 MBq, but three animals were injected with 9, 12 or 24 MBq 2-[^18^F]FDG, respectively. After 30, 60, or 120 min, the mice were sacrificed by cervical dislocation before the respective organs or tumors were harvested, cut into smaller pieces, briefly washed, and dabbed dry. The tissues were snap-frozen over liquid nitrogen to quench metabolism and stored on dry ice before analysis. Blood was withdrawn directly from the murine heart, cooled, and centrifuged (3000 g, 15 min, 4°C) to gain plasma. In summary, blood, brain, heart, kidney, liver, lung, HT1080 and HT29 tumors were collected for gamma counter measurements (2480 Automatic Gamma counter, Wizard^2^3, PerkinElmer) and subsequent radiometabolite analysis with HPLC. As each HPLC run lasted 35 min, only three organs (or two organs + one tumor) were harvested per mouse.

In addition, five mice underwent a 120 min dynamic 2-[^18^F]FDG scan with an Inveon μPET/CT device (Siemens), applying the same dose as stated before. Animals were placed in the scanner feet first in prone position for 120 min (29 frames, frame duration 5–600 s), restrained on a heating pad (38°C) with PEHA adhesion, and covered with a custom-made blanket. Heat pad temperature and respiratory rate were monitored with a BioVET CT1 system (Siemens). Concerning anesthesia, a range of 2 L/min 2% isoflurane in oxygen (induction) and 0.5 L/min 1% isoflurane in oxygen (maintenance during the scan) was used. For image generation and quantification PMOD Software (Fuse it tool, Version 3.8, RRID:SCR_016547) was used. For PET, the reconstruction algorithm OSEM3D/MAP was applied (MAP subsets: 16, iterations: 18, beta-value: 0.0527972, ordinary Poisson type OSEM3D/MAP, voxel size = x:0.388 mm, y:0.388 mm, z: 0.796 mm; image size = x:256, y:256, z:159). For CT, Feldkamp cone beam reconstruction was used (voxel size = 0.0975 mm x/y/z, image size = 1024 x/y/z). PET images were decay-corrected, attenuation-corrected (CT-based), scatter-corrected, dead-time-corrected and the detector normalized.

Concerning the volumes of interest (VOIs), the whole tissue was delineated in the case of the brain and the lung. In the case of the kidney, the pelvis was excluded. For the liver, three small representative VOIs were placed across the organ and averaged, thereby avoiding falsification by large blood vessels and heterogeneity. As for the heart, the muscle was delineated, thereby excluding the large blood pool.

For image quantification, no smoothing was applied. PET images were co-registered (trilinear interpolation) to the CT image (reference, with reduction 2/2/2 x/y/z, final voxel size = 0.195 mm x/y/z, image size = 512 x/y/z) using automated rigid matching or manual re-slicing in case of unsuccessful automated matching. CT windowing: 90–500 HU.

#### *In vitro* knockdown of oxPPP enzymes

HT29 cells were transfected using 12.5 nM of G6PD, H6PD, or non-targeting SMARTpool ON-TARGETplus siRNA, DharmaFECT transfection reagent No. 1 (all Horizon, PerkinElmer) and Opti-MEM (Gibco, ThermoFisher), following the provider’s protocol. The optimal knockdown was found to be reached 72 h after transfection (Figure S1). Thus, all experiments were started 72 h after siRNA application. Successful and selective knockdown was monitored with WB analyses (Figures 7A and S2).

#### *In vivo* knockdown of oxPPP enzymes

Additionally, intratumoral *in vivo* silencing of G6PD/H6PD in Fox Chase SCID Beige mice was tested.

For *in vivo* silencing of G6PD or H6PD, the siRNA pools for *in vitro* use were tested beforehand with HT29 cells to define the most active siRNAs from the pools (Figure S3A). HT29 xenografts were grown as stated before. Injection of siRNA was performed as soon as tumors were clearly palpable and considered big enough for a safe injection of 50 µL. Before injection, the respective *in vivo*-grade siRNAs (No. 19/20 for G6PD, No. 10/11 for H6PD, No. 6 non-targeting) were prepared and complexed with *in vivo*-jetPEI (Polyplus) according to the suppliers’ instructions. The mice were briefly anesthetized with isoflurane and 50 μL containing 10 μg siRNA (N/P ratio 8) were injected directly into the tumor from different angles. For the first experiments, mice were sacrificed after 48 or 72 h (based on our *in vitro* experiments) and tumors, colon, and liver (as reference organs to exclude incorrect injection) were harvested as previously stated. As the siRNA mixture was spiked with 1 μg fluorescently labeled non-targeting siRNA, pictures of the whole mouse, the sliced tumor and two reference organs were taken postmortem with an optical imaging device (IVIS, RRID:SCR_020397) to ensure successful injection. To assess protein silencing, WB were performed with the tumor lysates as described below. In contrast to the cell experiments, analyses were performed with another H6PD antibody (1:1000 Proteintech Cat# 15255-1-AP, RRID:AB_10642699) but later repeated with the abcam antibody mentioned in the section describing western blot analysis. In addition to the single injection, double injection within 3 days, as well as double siRNA amount and longer incubation time (96 h for G6PD only) were evaluated. The latter approach was based on a publication for G6PD knockdown in brown adipose tissue.^61^ However, we could not reach a significant downregulation of oxPPP enzymes (Figure S3).

#### 2-[^18^F]FDG accumulation experiments

HT1080 and HT29 cells were incubated with 1 MBq/mL 2-[^18^F]FDG for 1 h in MEM (1 g/L glucose), and accumulation was determined as previously published.^2^ Briefly, 300.000 or 700.000 cells in 2 mL MEM or RPMI, respectively, were seeded in 6-well plates two days prior the experiment. On the day of the experiment, the culture medium was removed and cells were washed once with MEM, before 1.5 MBq 2-[^18^F]FDG in 1.5 mL MEM were added. The same amounts of medium and 2-[^18^F]FDG were also applied to a triplicate of cell-free wells as reference. After 1 h in the incubator, 100 μL of each reference well were withdrawn and put into separate Eppendorf tubes. Then, the supernatant of the wells was removed (including the reference wells) and all wells were washed two times with 1 mL PBS. The cell wells were then incubated at 37°C with 500 μL Accutase (Gibco) until the cells were detached before subsequently, 1 mL MEM was added and the cell suspension was mixed thoroughly. Immediately after mixing, 100 μL of each cell suspension were transferred into Eppendorf tubes for gamma counting. The reference wells were filled up with 1.5 mL PBS and 100 μL were derived again to determine the residual radioactivity after washing (blank). After measuring the samples with a gamma counter (PerkinElmer), the cell content of each Eppendorf tube was determined with a LUNA automated cell counter (logos) using trypan blue.

For the knockdown experiments, HT29 cells were also incubated with 2-[^18^F]FDG as stated above, 72 h after siRNA addition. To assess the effects of oxPPP enzyme inhibitors, 100 μM DHEA (Sigma-Aldrich), 84 (HT1080) or 130 μM (HT29) CBX (Sigma-Aldrich), or 50 μM G6PDi-1 (kindly provided by the Rabinowitz-Lab^46^) were added either 1 h before (all 3 inhibitors) or simultaneously with 2-[^18^F]FDG (DHEA, G6PDi-1). CBX concentration was based on previous analyses of IC50 concentrations, DHEA concentration was chosen according to literature^46^^,^^62^^,^^63^ and G6PDi-1 concentration was based on recommendations by the research group providing the inhibitor.

#### Radiometabolite detection with HPLC

To study 2-[^18^F]FDG metabolism and its intermediates over time, radiometabolites were determined at the time-points 30, 60 and 120 min for murine organs, tumors and both cell lines as previously described.^2^ Before analysis, fresh frozen tissue was homogenized with an ULTRA-TURRAX (Ika) in 3:1 methanol and PBS (approximately 2 mL/100 mg tissue) on ice and shortly sonicated. The cultivated cells were scraped off and homogenized correspondingly. Methanol for lysis was preferred over other agents like perchloric acid as used by Rokka et al.,^5^ because it is less harsh and part of the used HPLC solvents. Cell or tissue lysates were then centrifuged for 4 min (4°C, 13,684 g). 100 μL of each supernatant were injected into a Shimadzu HPLC system via a cooled auto sampler. For radiometabolite analysis, an anion-exchanger Partisil 10 SAX column with 250 mm × 4.6 mm (Supelco analytical) and a gradient of 0.6 M sodium dihydrogen phosphate buffer with 3% methanol and 3% methanol in water (1 mL/min flow, 35 min run time) were used (see supplemental information for the gradient). Radio-peaks were detected with a Ramona∗ radioactivity-HPLC flow detector (Elysia Raytest) and all radio-peaks with an area at least five times larger than a corresponding background area were quantified (all radiopeaks detected = 100%).

To additionally analyze the influence of the oxPPP inhibitors and enzyme knockdown on the metabolism of 2-[^18^F]FDG, cells were treated with the respective inhibitor (HT1080, HT29) or siRNA (HT29) as stated above and metabolism was analyzed 1 h after 2-[^18^F]FDG addition, analogous to accumulation experiments.

#### Western blot analysis

For WB analysis, RIPA-buffer (ThermoFisher) was used to lyse cells in the presence of 1x protease inhibitor (Sigma-Aldrich). Cells were scraped off, shaken on ice for 30 min and subsequently centrifuged for 20 min at 4°C and 13,684 g. The protein concentration of each lysate was determined using a bicinchoninic acid kit (ThermoFisher). Samples were loaded into 4–20% Mini-PROTEAN TGX Precast Protein Gels (Bio-Rad) for gel electrophoresis. Then, semi-dry blotting was performed using nitrocellulose blotting membranes (Amersham) and a Trans-Blot Turbo Transfer System (Bio-Rad). Membranes were subsequently blocked with 5% dry milk powder in Tris-buffered saline with 0.1% tween 20 for 1.5 h at room temperature (RT). Thereafter, membranes were cut into two-halves to separately incubate them with a primary antibody overnight at 4°C against the respective enzyme (1:1000 anti-G6PD Abcam Cat# ab210702, RRID:AB_2923527, or 1:1000 anti-H6PD, Abcam Cat# ab170895, RRID:AB_2864314), or 1:2500 anti-β-actin (Abcam Cat# ab8227, RRID:AB_2305186). The next day, membranes were washed thoroughly three times and the secondary antibody was applied 1:2500 for 1 h at RT (Thermo Fisher Scientific Cat# A16104, RRID:AB_2534776). After washing the membranes again, protein bands were detected using the Clarity Western ECL Substrate and a ChemiDoc detection system (both Bio-Rad, RRID:SCR_021693). Protein bands of the target enzymes were normalized to β-actin using Fiji software (RRID:SCR_002285).

#### Enzyme activity assays

For photometric analyses, HT29 cells were scraped off in a mixture of 50 mM Tris buffer (pH 8), 1 mM EDTA, 0.05% Triton X-100 and 1x EDTA-free cOmplete/PhosSTOP (all Sigma-Aldrich), sonicated for 3x 50 seconds and subsequently treated as described for WB analysis. Samples were kept at −80°C until the measurement. Enzyme activity assays were carried out with a Hitachi U-2900 spectrophotometer based on a previously described protocol.^64^ Briefly, G6PD/H6PD activity was determined for 5 μg of crude protein in a 50 mM Tris buffer (pH 7.8) with 6.5 mM MgCl_2_ and 0.6 mM NADP^+^ (all Sigma-Aldrich). Immediately after the addition of 30 mM of the substrates G6P (Sigma-Aldrich) or FDG6P (Biosynth Carbosynth), NADPH formation was measured at 340 nm for 5 min at RT with or without addition of 100 μM DHEA.

### Quantification and statistical analysis

Student’s *t* test and one-way ANOVA were performed in GraphPad Prism (Version 7.03, RRID:SCR_002798), the calculation of normality distribution (Shapiro-Wilk test) and correlation using Spearman correlation was carried out with IBM SPSS statistics (Version 27, RRID:SCR_019096). A *P* of ≤0.05 was deemed significant and all values are given as mean ± SD. Asterisks report statistical significance: ∗p ≤ 0.05, ∗∗p ≤ 0.01, ∗∗∗p ≤ 0.001, ∗∗∗∗p ≤ 0.0001. The number of animals, organs, or technical replicates of cell experiments is indicated as “*n*” in the results section and figure captions. *In vitro* accumulation experiments were performed in dupli- or triplicates. Metabolism beyond 2-[^18^F]FDG-6-P *in vitro* was analyzed from dupli- or triplicates, but also single measurements were performed when n > 4.

## Data Availability

• Data reported in this paper will be shared upon request to the lead contact. • This study did not generate new code. • Any additional analysis information for this work is available by request to the lead contact.
