# Supplementary material for: A fingerprint of 2-[18F]FDG radiometabolites – How tissue-specific metabolism beyond 2-[18F]FDG-6-P could affect tracer accumulation
Source: iScience. 2023 Oct 6;26(11):108137. doi: 10.1016/j.isci.2023.108137 (PMC10585399; doi:10.1016/j.isci.2023.108137)
Supplement: Document S1. Figures S1–S3, Tables S1, and S2 [file mmc1.pdf]

iScience, Volume *26*

Supplemental information

A fingerprint of 2-[^{18}F]FDG radiometabolites – How tissue-specific metabolism beyond 2-[^{18}F]FDG-6-P could affect tracer accumulation

Eva-Maria Patronas, Theresa Balber, Anne Miller, Barbara Katharina Geist, Antje Michligk, Chrysoula Vraka, Maximilian Krisch, Nataliya Rohr-Udilova, Arvand Haschemi, Helmut Viernstein, Marcus Hacker, and Markus Mitterhauser

Supplemental information

Time (min)	% A (0.6 M sodium dihydrogen phosphate buffer with 3 % methanol)	% B (3 % methanol in water)
0 - 12	5	95
13	10	90
14 - 18	15	85
19 - 32	50	50
34 - 35	5	95

Table S1. Gradient profile of the HPLC runs, related to Figure 1.

Gene	H6PD	G6PD
**Unweighted_meta-Z_of_all_cancers**	**-3.028058243**	**5.104754536**
Adrenocortical_cancer	-0.224	-1.307
Bladder_cancer	-1.095	1.584
Brain_cancer_Astrocytoma	2.315	1.174
Brain_cancer_Glioblastoma	-0.836	0.638
Brain_cancer_Glioma	1.687	2.104
Brain_cancer_Medulloblastoma	0	0
Brain_cancer_Meningioma	-0.92	0.81
Brain_cancer_Neuroblastoma	-4.873	7.427
Breast_cancer	-5.307	4.185
Colon_cancer	-0.265	-0.594
Gastric_cancer	-0.638	1.069
Germ_cell_tumors	0.531	-1.269
Head_and_neck_cancer	0.27	0.841
Head_and_neck_cancer_Hypopharyngeal_cancer	-0.123	0
Head_and_neck_cancer_Oesophageal_cancer	-0.548	0
Head_and_neck_cancer_Oral_SCC	0.848	0.205
Hematopoietic_cancer_AML	-1.654	1.814
Hematopoietic_cancer_B_ALL	-0.273	0.442
Hematopoietic_cancer_Burkitt_lymphoma	0.668	-0.29
Hematopoietic_cancer_CLL	0.072	1.544
Hematopoietic_cancer_DLBCL	0.477	0.603
Hematopoietic_cancer_FL	-2.66	1.595
Hematopoietic_cancer_Mantle_cell_lymphoma	0.771	2.301
Hematopoietic_cancer_Multiple_myeloma	-0.103	2.832
Kidney_cancer	-0.209	0
Liver_cancer	-1.438	-0.824
Liver_cancer_Primary	-0.049	-0.255
Lung_cancer_ADENO	-0.861	2.156
Lung_cancer_LCC	-0.319	0.343
Lung_cancer_SCC	-1.922	1.712
Lung_cancer_SCLC	0.258	0
Melanoma	1.114	0.319
Melanoma_Metastasis	1.127	1.193
Mesothelioma	-0.479	0
Ovarian_cancer	-0.992	-1.996
Pancreatic_cancer	-1.207	0.693
Prostate_cancer	-1.005	-0.014
Sarcoma_Ewing_sarcoma	-0.375	0.446
Sarcoma_Osteosarcoma	-0.674	0.397

Colour	Meaning
(green)	higher expression is associated with longer survival
(magenta)	higher expression is associated with shorter survival

Source: PRECOG database Stanford 2021

Table S2. Relationship between G6PD/H6PD gene expression and survival across different cancer types, related to Figure 5.

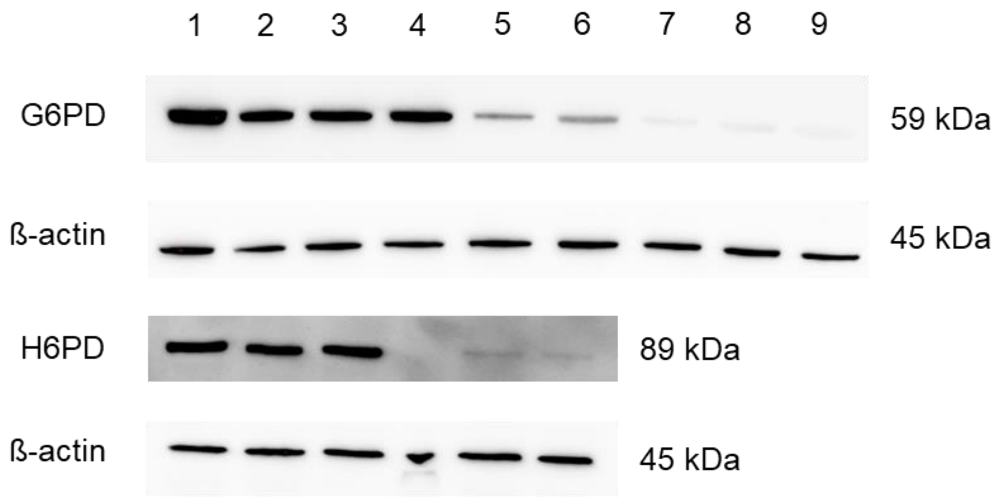

Amount of siRNA applied and time course of silencing:

G6PD

1: control
2: 12.5 nM, 24 h
3: 25 nM, 24 h
4: 50 nM, 24 h
5: 12.5 nM, 48 h
6: 50 nM, 48 h
7: 12.5 nM, 72 h
8: 25 nM, 72 h
9: 50 nM, 72 h

H6PD

1-3: control
4-6: 12.5 nM, 72 h

Figure S1. Western blots of preliminary tests with SMARTpool ON-TARGETplus siRNAs to find the optimal concentration for G6PD/H6PD knockdown in HT29 cells, related to Figure 7.

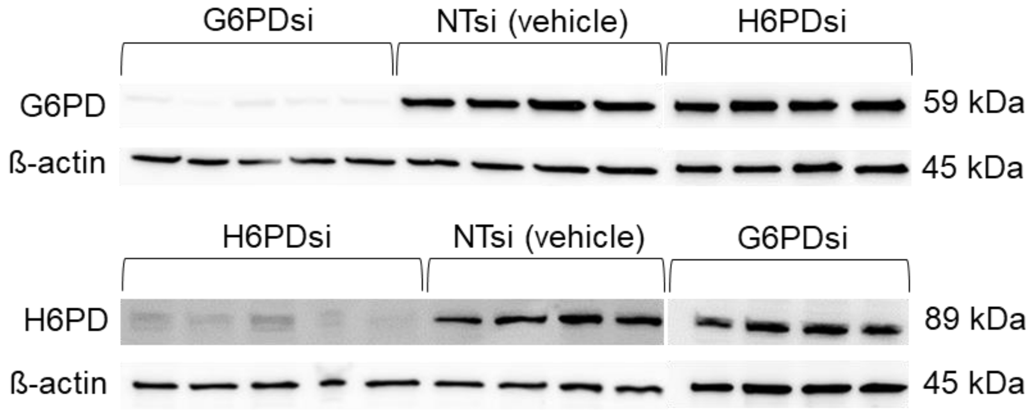

Figure S2. Representative western blots that exclude compensatory overexpression of the respective other enzyme after knockdown with siRNA (samples from different experiments), related to Figure 7.

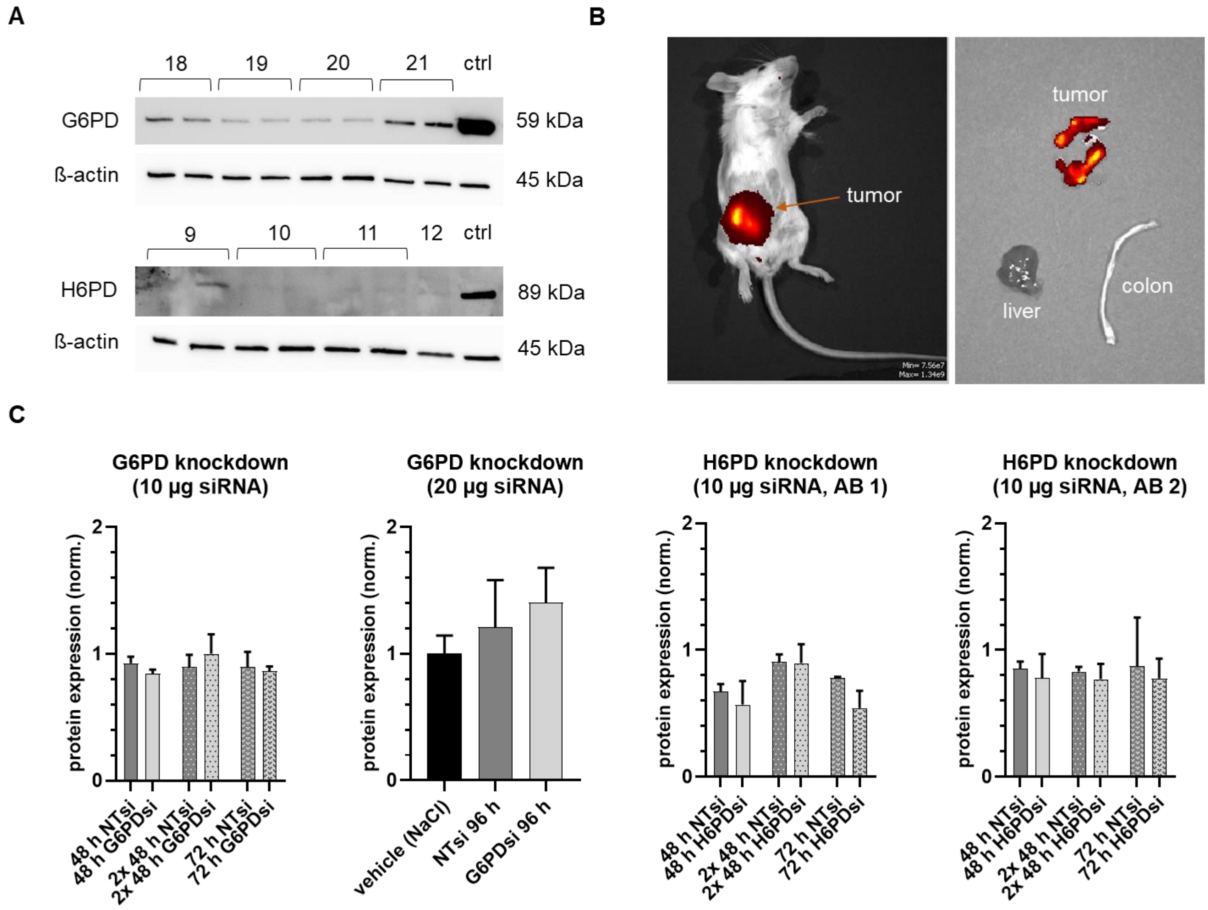

Figure S3. Attempts for *in vivo* knockdown of G6PD/H6PD were not successful, related to Figure 7. (A) Western blot analysis determining the most effective siRNAs within the tested pool. (B) Post-mortem IVIS® images showing successful siRNA injection and good distribution in HT29 tumor, and (C) summary of western blot analyses of G6PD/H6PD knockdown (*n* = 3 each, NTsi = non-targeting siRNA, AB 1 = proteintech antibody, AB 2 = abcam antibody). Bar graph data are represented as mean ± SD.
